# Signal Transduction in Cereal Plants Struggling with Environmental Stresses: From Perception to Response

**DOI:** 10.3390/plants11081009

**Published:** 2022-04-07

**Authors:** Małgorzata Nykiel, Marta Gietler, Justyna Fidler, Beata Prabucka, Anna Rybarczyk-Płońska, Jakub Graska, Dominika Boguszewska-Mańkowska, Ewa Muszyńska, Iwona Morkunas, Mateusz Labudda

**Affiliations:** 1 Department of Biochemistry and Microbiology, Institute of Biology, Warsaw University of Life Sciences-SGGW, 02-776 Warsaw, Poland; marta_gietler@sggw.edu.pl (M.G.); justyna_fidler@sggw.edu.pl (J.F.); beata_prabucka@sggw.edu.pl (B.P.); anna_rybarczyk_plonska@sggw.edu.pl (A.R.-P.); jakubgraska1@gmail.com (J.G.); mateusz_labudda@sggw.edu.pl (M.L.); 2 Plant Breeding and Acclimatization Institute-National Research Institute, 05-870 Radzików, Poland; d.boguszewska-mankowska@ihar.edu.pl; 3 Department of Botany, Institute of Biology, Warsaw University of Life Sciences-SGGW, 02-776 Warsaw, Poland; ewa_muszynska@sggw.edu.pl; 4 Department of Plant Physiology, Poznań University of Life Sciences, Wołyńska 35, 60-637 Poznań, Poland; iwona.morkunas@gmail.com

**Keywords:** abiotic stress, biotic stress, cereal, crosstalk, drought, heavy metal, phytohormone, salinity, pathogen, pest

## Abstract

Cereal plants under abiotic or biotic stressors to survive unfavourable conditions and continue growth and development, rapidly and precisely identify external stimuli and activate complex molecular, biochemical, and physiological responses. To elicit a response to the stress factors, interactions between reactive oxygen and nitrogen species, calcium ions, mitogen-activated protein kinases, calcium-dependent protein kinases, calcineurin B-like interacting protein kinase, phytohormones and transcription factors occur. The integration of all these elements enables the change of gene expression, and the release of the antioxidant defence and protein repair systems. There are still numerous gaps in knowledge on these subjects in the literature caused by the multitude of signalling cascade components, simultaneous activation of multiple pathways and the intersection of their individual elements in response to both single and multiple stresses. Here, signal transduction pathways in cereal plants under drought, salinity, heavy metal stress, pathogen, and pest attack, as well as the crosstalk between the reactions during double stress responses are discussed. This article is a summary of the latest discoveries on signal transduction pathways and it integrates the available information to better outline the whole research problem for future research challenges as well as for the creative breeding of stress-tolerant cultivars of cereals.

## 1. Introduction

The climatic conditions have changed many times during the history of Earth, but now, these alterations are strongly intensified by heavy industrial activity. As agriculture is a branch of the economy most dependent on climatic conditions, the progressing climate changes create a completely new situation for agricultural activity, especially for plant production [1]. The rate of temperature changes causes many extreme atmospheric phenomena that have not occurred or have appeared very rarely so far. Noticeable changes in the quantity and quality of rainfall (an increase in the number of storms followed by periods without rainfall) increase the risk of both flooding and drought. In addition, the increase in temperature may favour the overwintering of plant pathogens and pests, which have not so far posed a threat to native crops [2]. Moreover, in the era of global warming, the mobility of pollutants, including heavy metals in the environment increases [3].

Plant responses to environmental factors are extraordinarily complex. They can be observed at various levels of plant organisation, ranging from changes at the cell level, i.e., the changes in the activity of basic biochemical processes such as DNA replication, respiration, and photosynthesis to morphological and anatomical changes in plant organs [4,5,6,7]. However, mentioned biochemical changes are preceded by the activation of an efficient signalling system that endures environmental fluctuations [8]. The presented review highlights issues related to stress factor recognition/stress factor perception, induction, and transmission of the signal, and subsequent signalling responses at molecular and metabolic levels in cereals under the influence of various stress factors caused by global warming. This work shows the latest research results in the context of the defence mechanism induction of cereals to different abiotic stress and tolerance/existence under stressful environments.

It is well known that there are four main common signal transduction pathways in plants, which can interact with each other, i.e., reactive species signalling, calcium-dependent signalling, plant hormone signalling and signalling based on phosphorylation/dephosphorylation of proteins by kinase cascades [9]. Plant hormones are important regulators of the tailored responses to different stresses. The coordination of regulatory mechanisms among different hormones, or the interaction of hormone signalling with other molecules, such as reactive oxygen species (ROS), reactive nitrogen species (RNS), and hydrogen sulphide (H_2_S), is flexible and changes over time [10,11,12].

Abiotic and biotic stressors can cause a state of excess excitation energy in plant cells which leads to universal consequences: disturbances in electron transport, increased reduction in plastoquinone and uncontrolled generation of ROS, mainly superoxide anion (O_2_^−^), hydrogen peroxide (H_2_O_2_), hydroxyl radical (HO^−^) and singlet oxygen (_1_O^2^). The reaction of ROS with nitric oxide leads also to the formation of RNS, and potentially the appearance of not only oxidative but also nitrosative stress [13]. As ROS and RNS can act as a double-edged sword, they also play an important role in redox signalling as secondary messengers or signalling molecules and take part in signal transmission to the nucleus through redox reactions using the mitogen-activated protein kinases (MAPK) pathway and control of the antioxidant system in the plant cell [10]. Emerging signals are also facilitated by a series of phosphorylation/dephosphorylation events. This protein modification can both activate or deactivate effective proteins, which adjust cell metabolism to current environmental conditions with low energy costs. Phosphorylation cascade can be calcium-dependent (calcium-dependent protein kinases; CDPKs, calcineurin B-like interacting protein kinase; CIPK) or calcium-independent, for example, MAPK.

All signalling pathways can lead to the activation of appropriate transcription factors (TFs), enabling the transcription of genes crucial for maintaining plant homeostasis under stress. Additionally, the gene expression may be regulated by the presence of microRNA (miRNAs)-key regulators of plant responses to abiotic and biotic stresses and plant development [14]. The end result is the translation of proteins whose role is to reduce stress (e.g., by sequestration of salt ions or inactivation of heavy metals) or to eliminate its negative consequences. The resulting metabolic adjustments are crucial for maintaining the balance between simultaneously occurring processes of growth and development and stress defence. In this review, we present, confront and discuss different recent views on signal transduction in cereal plants under various stresses.

## 2. Drought

During drought, disturbances in the water management of plants occur, leading, inter alia, to closing the stomata and limiting transpiration [15]. Overlapping structural and functional changes, including oxidative stress, inhibition of photosynthesis and alternations in the distribution of assimilation products lead to disruption of plant growth and development [5,16]. Under drought, assimilates move from leaves (donor organs) to the roots (acceptor organs), which are responsible for water and nutrient uptake, at the expense of the biomass of the aerial parts. Plants suffering from water deficit are usually smaller, light-coloured, lack turgor, and are more susceptible to disease and pest attacks. All of the above result in lesser nutrients supplementation, which reduces the yield of a crop [1].

Plants initially identify water scarcity conditions by the roots, which results in the initiation of several molecular signals that transfer from the roots to the shoots [17]. For this reason, the root is a key organ that determines the effectiveness of the plant’s response to the stress of water shortage. The root shows high plasticity of its features and its reaction to drought can vary. There can be observed root elongation [18] or shortening [19], but also its length may not change [20]. The root system of cereals varies, e.g., wheat (*Triticum aestivum* L.) produces coarser, moderately branched roots, which allows for more efficient water management, while rice (*Oryza sativa* L.) forms thin, more branched underground organs which can better penetrate soil [21].

A special role in signalling is attributed to chloroplasts and mitochondria that are considered sensors of changes occurring in the environment. Chloroplasts and mitochondria generate ROS and transmit retrograde signals to the nucleus [22]. The direct signals of drought are transduced in plants through ROS, such as singlet oxygen (^1^O_2_), superoxide anion radical (O_2_**^·−^**), hydroxyl radical (HO**^·^**) and hydrogen peroxide (H_2_O_2_) [23]. Signalling by ROS can take place through pathways based on susceptible proteins containing thiol groups which are subject to reversible oxidation [24]. Thiol reducing molecules, such as glutathione and specific isoforms of thiols reductases, thioredoxins (TRX) and glutaredoxins (GRX), were found in diverse nuclear subcompartments, further supporting the assumption that thiol-dependent systems are active in the nucleus [25]. Thioredoxins and glutaredoxins are not only responsible for the reduction of thiol groups of numerous metabolic enzymes and molecules belonging to ROS scavenging systems, but also regulate thiol-based post-transcriptional redox modifications of proteins [26]. In *T. aestivum*, TRX isoforms are accumulated in the nucleus upon oxidative stress. It is likely that the influence of ROS on the expression of nuclear genes may be based on the regulation of redox-sensitive TFs [27].

The MAPK cascade-mediated ROS removal is an important mechanism regulating drought stress tolerance [28]. The MAPK cascade leads to the activation of antioxidant enzymes such as superoxide dismutases (SOD), peroxidases (POX) and catalases (CAT) in many cereal plants [29,30]. A proteomic study of *T. aestivum* plants revealed the abundance of CAT and three isoforms of SOD (chloroplastic cytosolic Cu/Zn-SOD and mitochondrial Mn-SOD) in response to drought. These antioxidant enzymes were involved in the survival strategy of wheat by avoiding the excess generation of ROS [31]. Moreover, salicylic acid (SA), ethylene (ET), jasmonic acid (JA), cytokinins (CKs), gibberellins (GAs) and brassinosteroids (BRs) play a vital function in regulating various phenomena in cereal acclimatisation to drought stress [32]. For example, BR signalling regulates drought tolerance in wheat, which is partially achieved through brassinazole-resistant 2 (TaBZR2) TF. TaBZR2 increased the *glutathione S-transferase-1* (*TaGST1*) expression and a decrease in ROS level was observed [33]. Furthermore, maize calcium/calmodulin-dependent protein kinase (ZmCCaMK) was involved in BR signalling and it was required for BR-induced antioxidant defence [34].

The key step in cereals’ response to drought is increased concentrations of abscisic acid (ABA) in the root, which may contribute to increased root hydraulic conductivity. By this mechanism, cereals adjust their cellular processes by triggering a network of long-distance signalling events that start with the perception of stress signals and lead through transduction of those signals to switch on acclimation cellular responses, such as changes in gene expression [35]. ABA regulates the expression of different stress-responsive genes involved in the accumulation of compatible osmolytes, synthesis of late embryogenesis abundant (LEA) proteins, dehydrins, chitinases, glucanases, as well as other protective proteins, such as the heat shock protein (HSP) [36]. The resulting osmotic adjustment helped to maintain higher leaf relative water content at low leaf water potential under drought. It enabled sustained growth while under reduced leaf water potential [37]. Recent evidence indicates that H_2_S is actively involved in the regulation of ethylene-induced stomatal closure and also interacts with H_2_O_2_ by impacting the activities of the inward K^+^ ion and anion channels [38]. Wheat can adapt to osmotic stress by H_2_S production and activation of the antioxidant system [12]. It was proven that H_2_S induced the ABA-triggered ascorbate–glutathione (AsA-GSH) cycle under osmotic stress. Obviously, H_2_S was involved in the ABA-related closing of stomata in response to various environmental stresses, however, the interaction between them is still unclear and requires further research [11].

In response to osmotic stress occurring often with drought, levels of growth-stimulating hormones: GAs, CKs, sometimes indole acetic acid (IAA) decrease, while there is an observed increase in the level of hormones that usually inhibit cell elongation growth or accelerate maturation and/or aging of tissues: ABA, ET, JA, methyl jasmonate (MeJA) and BRs [39]. Here, ABA acts as the hub of the hormonal crosstalk between several stress signalling cascades [40]. Osmotic stress-responsive gene expression is regulated by ABA-dependent and ABA-independent pathways [41]. In the ABA-dependent pathway, numerous types of TFs, such as MYeloBlastosis (MYB), a basic helix–loop–helix (bHLH), the basic region leucine zipper (bZIP), ethylene response factor (ERF) and homeodomain TF are involved [42]. It was proven that overexpression of *JERF1* (ERF gene) significantly enhanced drought tolerance of transgenic rice [43]. According to Zhang et al. [43], the JERF1 activated the expression of stress-responsive genes and increased the synthesis of the osmolyte proline by regulating the expression of *OsP5CS*, encoding deltal-pyrroline-5-carboxylate synthetase (proline biosynthesis enzyme) [43]. JERF1 also triggered the expression of two rice genes encoding ABA biosynthesis enzymes, zeaxanthin epoxidase 2 (*OsABA2*) and xanthoxin dehydrogenase (*Os03g0810800*) [43].

Signal transduction of osmotic stress also depends on Ca^2+^, nitric oxide (NO), reactive sulphur species which induce MAPK [28], calcium-dependent protein kinase [44], and calcineurin B-like interacting protein kinase families of protein kinases [45], or phospholipid signalling [13]. The MAPK cascade plays an important role in the drought stress response mainly by responding to ABA and regulating ROS production. Numerous components of MAPK cascades were described as responding to water deficiency in cereals. For example in rice, the transcripts of *OsMKK4*, *OsMKK1*, *OsMPK8*, *OsMPK7*, *OsMPK5* and *OsMPK4* were accumulated under drought stress [46,47,48]. In wheat, the expression levels of *TaMKKK16*, *TaMKK1* and *TaMPK8* changed in response to drought stress [49]. Ma et al. [50] found that *OsMKK10.2–OsMPK3* were responsible for conferring drought stress tolerance in rice via ABA signalling [50]. However, the exact relationship of the MAPK cascade with ABA has not yet been described [28]. In rice, MAPK5, MAPK7, MAPK8 and MAPK12 were induced by drought and MAPK4 was repressed under water shortage [51]. In rice, MAPK kinases regulated the activity of transcription factors such as OsWRKY30 and increased drought tolerance [52].

The CDPKs and CIPK are families of protein kinases. In rice, a Ca^2+^ dependent kinase such as OsCIPK12 increased the concentration of proline and soluble sugars, which may improve drought tolerance. Additionally, OsCDPK7 enhanced the expression of the gene whose product is the rab16A protein, potentially involved in drought tolerance [53]. In addition to the mentioned protein kinases, rice has a total of 74 heat shock proteins classified into four categories: sHSP, HSP70, HSP90 and HSP100 [54]. These HSPs are activated by ABA-dependent heat shock transcription factors (HSFs), but only some of them are activated by drought [55]. Furthermore, in wheat and barley, the expression of several dehydrins (Dhn) belonging to group two of LEA proteins was observed under drought [56]. Karami et al. [57] reported induction of several genes of Dhn, such as *Dhn1*, *Dhn3*, *Dhn5*, *Dhn7*, and *Dhn9*, in barley flag leaf under drought. Relative expression levels of *Dhn3* and *Dhn9* revealed positive correlations with chlorophyll a and b contents, osmotic adjustment, plant biomass and grain yield, and negative correlations with malondialdehyde (MDA), a marker of membrane oxidative lipids damage, and electrolyte leakage levels.

The majority of LEA proteins display a preponderance of hydrophilic and charged amino acid residues. On the basis of the literature, their function as antioxidants, membranes and protein stabilisers, and indirect participants as molecular shields in cell protection are considered [58]. *HVA1* gene encoding group three of LEA protein from barley (*Hordeum vulgare* L.) was transformed into rice and the tolerance to water deficit of the transgenic rice was improved under the greenhouse conditions [59]. The overexpression of the *HVA1* gene in the roots and leaves of wheat also tended to retain tolerance to drought stress. In wheat, in response to drought, the size of LEA proteins reached up to 200 kDa, therefore, these proteins were resistant to denaturation [60]. It was observed that overexpression of *OsLEA6* and *OsLEA3-1* led to enhanced drought tolerance of rice plants in the field [61].

As an important metabolic pathway, phosphatidylinositol metabolism generates signalling molecules that are essential for survival under drought [46]. Phospholipid molecules are involved in signalling processes leading to adjustments in root growth, pollen and vascular development, hormone effects and cell responses to environmental stimuli in plants [46]. Wang et al. [62] showed that the expression of maize *ZmPLC1*, encoding phospholipase C, was up-regulated under dehydration and it improved the drought tolerance of maize through the interaction with other signalling pathways in guard cells [62].

It is well known that the NO performs the signalling function in plant cells. Signal transduction by NO is mediated by cyclic guanosine monophosphate (cGMP) and activation of guanylate cyclase [63]. NO regulates the levels of cellular ROS content and toxicity through the activation of antioxidant enzymes [64]. Gan et al. [64] showed that NO (applied exogenously) increased drought resistance in barley. The application of NO not only increased the activity of antioxidant enzymes but also increased the content of proline. NO was also found to crosstalk with ABA, JA, SA and CKs to mitigate the adverse effect of drought stress [65]. There are also many studies showing the cooperation of H_2_S and NO in response to drought [11].

## 3. Salinity

Salinity is one of the most important abiotic stress that negatively influences plant growth and productivity, especially rice, wheat and barley, which are the main food crops worldwide [66,67]. Soil salinity caused 25–30% of the irrigated area worldwide to be commercially unproductive and it is estimated that progressive salinity, expanding at a rate of 10% per year, will lead to nearly 50% of agricultural land by 2050 being unproductive [68]. The high concentration of salts in the soil may cause a reduction in water and nutrient uptake due to salt accumulation in the root zone (physiological drought), therefore inducing ion and nutrient imbalance, and water stress in plants. Salinity, due to the presence of NaCl in the soil, is the most common, hence the most harmful effect of salinity is the accumulation of Na^+^ and Cl^–^ [69]. Excess Na^+^ in the plant inhibits the uptake of essential micronutrients such as K^+^ and Ca^2+^ from the soil, a shortage of the second one is especially crucial because it participates in the maintenance of cell membrane integrity, as well as in the synthesis of new cell walls [70]. Thus, overaccumulation of Na^+^ leads to damage and enhanced permeability of membranes. Loss of membrane integrity can also lead to K^+^ leakage from cells, which can affect enzymatic reactions since many enzymes require K^+^ as a cofactor. Additionally, these enzymes are sensitive to high cytosolic Na^+^ content. The accumulation of Na^+^ also alters the activity of photosynthetic enzymes and it is harmful to other photosynthesis compounds, such as chlorophylls, and carotenoids [71]. What is more, it is assumed that disturbed ion homeostasis (excess of Na^+^ and shortage of Ca^2+^ and K^+^) might contribute to oxidative stress which is resulting in the overproduction of ROS and an inefficient ROS detoxification system. The following consequences are oxidative damage of various plant cellular components such as nucleic acids, proteins, sugars and lipids, and hence the inhibition of proper plant development and growth [72].

The response of plants to salinity occurs through the perception and transduction of a signal associated with the disruption of ion and osmotic homeostasis. It is considered that plant cells sense the increase in cytosol Na^+^ levels through a sensor or a receptor. Nonetheless, no specific sensor or receptor was identified in plants so far. Therefore, it is not known how an excess of Na^+^ is detected by plants, so it can be assumed that the perception of salt stress signal remains unrevealed [73]. However, the most common salt stress signalling pathways—the salt overly sensitive (SOS)—are well characterised in plants, including cereals. Additionally, the MAPK cascade, which transduces stress signals to a variety of transcription factors that further activate salt-responsive genes, plays an important role in salt stress signalling in plants [74].

**Figure 1:**
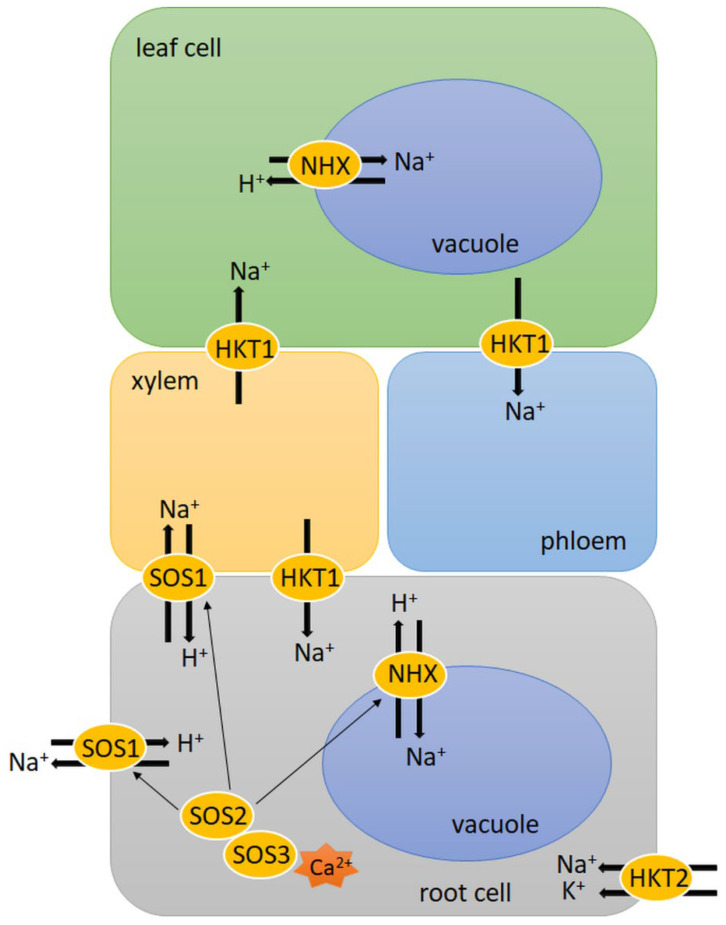
Na^+^ transportation under salinity. Plants remove Na^+^ from the cytoplasm using plasma membrane Na^+^/H^+^ antiporter (SOS1), which is activated through phosphorylation, catalysed by the SOS2–SOS3 kinase complex, SOS3 is a Ca^2+^ sensor. Compartmentation of Na^+^ into vacuoles occurs by Na^+^/H^+^ antiporter (NHX), which is also activated by SOS2–SOS3 kinase complex. High-affinity K^+^ transport (HKT) proteins, are Na^+^ transporters (class 1) or Na^+^/K^+^ symporters (class 2). HKT1 proteins remove Na^+^ from xylem. HKT2 play role in Na^+^ uptake in the root. Details are described in Salinity paragraph.

SOS pathway genes encode proteins that are engaged in the active efflux of excess Na^+^ from the cytosol (Figure 1). SOS1 is a plasma membrane Na^+^/H^+^ antiporter, activated through phosphorylation catalysed by the SOS2–SOS3 kinase complex. SOS3 is a Ca^2+^ sensor, which belongs to the calcineurin B-like signal protein family [75]. It perceives the cytosolic Ca^2+^ signal, which is triggered by a salt-induced excess of Na^+^. ABA plays a key role in increasing Ca^2+^ content, which is released from intracellular storage compartments [76]. Then, SOS3 interacts with SOS2, which is a CIPK serine-threonine protein kinase. The SOS3/SOS2 kinase complex regulates the expression of *SOS1* genes therefore it can stimulate SOS1 Na^+^/H^+^ antiporter activity [77]. In seedlings of three bread wheat genotypes, which were characterised as highly tolerant, moderately tolerant and sensitive to salinity stress, the expression of *SOS1*, *SOS2* and *SOS3* genes was observed at a significantly higher level in the salt-tolerant genotype. What is more, both constitutive and salt-induced expression of *SOS1* was 2-fold higher in the leaf of this genotype. This was correlated with low Na^+^ levels in tissue and better leaf K^+^/Na^+^ ratio in leaves, which was probably a result of the facilitated exclusion of toxic Na^+^ into root apoplast [75]. Similarly in experiments by Jiang et al. [77], the expression of several genes belonging to the TaSOS1 gene family was up-regulated in response to salinity in the wheat-tolerant genotype after 1 day of salt treatment. What is more, overexpression of the wheat genes encoding TaSOS1 and TaSOS1-974 (with a deletion on the C-terminus) in tobacco resulted in improved Na^+^ efflux and K^+^ influx rates in the roots of the transgenic plant compared to wild-type (WT) tobacco upon salt stress. Among these three types of plants, the lowest content of MDA and electrolyte leakage was observed in *TaSOS1-974* transgenic plants while the highest was observed in WT tobacco. This indicates that the overexpression of *TaSOS1-974* might alleviate oxidative damage of the plasma membrane generated upon salinity [78]. Comparable results were obtained in Arabidopsis SOS1 mutant plants with the overexpression of durum wheat (*Triticum durum* Desf.) gene *TdSOS1∆972* (with a deletion on the C-terminus). These plants showed greater water retention capacity and maintained a better K^+^/Na^+^ ratio in their shoots and roots, as well as their seeds, had a better germination rate upon salinity than in Arabidopsis *SOS1* mutant transformed with empty binary vector or *TdSOS1* (full-length) [79]. These results confirmed that in proteins belonging to the SOS1 family, the C-terminus function as an auto-inhibitory domain. Autoinhibition of SOS1 is released when the C-terminus domain is phosphorylated by activated SOS2 [80]. Additionally, in rice and barley, the involvement of SOS genes in response to salinity was observed. Fu et al. [81] showed that rice *OsSOS3* was significantly up-regulated in roots under salt stress. Additionally, the expression of *OsSOS2* and *OsSOS1* was markedly up-regulated and a high transcript level of these genes was maintained. In turn, barley *HvSOS3* was only slightly up-regulated in roots under stress. Other barley *SOS* genes, *HvSOS1* and *HvSOS3*, showed slight changes in roots during salt treatment. All tested rice genes showed higher absolute expression than barley genes. However, rice was more sensitive to salt stress than barley. In rice, a higher excess of Na^+^ was observed in the shoots, which was harmful for physiological processes, e.g., protein degradation. On the other hand, in rice, the level of Na^+^ in the roots was lower than in barley, which might be the result of Na^+^ efflux through the SOS pathway. Despite this phenomenon, barley maintains normal metabolism. These results show the differences in salt tolerance between these two species [81].

Besides the exclusion of Na^+^ from the cytosol, compartmentation of Na^+^ into vacuoles by tonoplast Na^+^/H^+^ antiporter (NHX) is also another essential mechanism in salt stress response (Figure 1). The necessary proton gradient required for NHX activity is derived from vacuolar H^+^-pyrophosphatase and H^+^-ATPase. The SOS3/SOS2 kinase complex regulates both NHX and H^+^-ATPase activity under salt stress. In wheat, the expression of the *NHX1* gene was markedly increased under saline conditions compared to the control [82]. Additionally, overexpression of the wheat *TaNHX2* gene in eggplant (*Solanum melongena* L.) and sunflower (*Helianthus annuus* L.) increased salinity tolerance in comparison to WT plants. Both transgenic species showed improved growth as well as reduced ROS and MDA contents, which correlated with the high activity of antioxidant enzymes such as SOD and ascorbate peroxidase (APX) [83,84]. A comparison of salt stress response in barley and rice showed that the expression of one of the *NHX* genes was significantly higher in barley (*HvNHX5*) than in rice (*OsNHX5*) in the roots treated with salt. However, the expression of rice *OsNHX1*, *OsNHX2* and *OsNHX4* in shoots was higher than in barley *HvNHXs*. This may indicate that a higher concentration of Na^+^ in rice shoots is a result of the up-regulated expression of NHX genes [81]. Moreover, the overexpression of barley *HvNHX2* in Arabidopsis showed that, under salt conditions, transgenic plants grew normally, while WT plants were not able to. Additionally, transgenic plants had a higher concentration of Na^+^ in the shoots and had longer roots than WT plants [85]. Similarly, the overexpression of *OsNHX1* in transgenic rice showed increased salt tolerance in transgenic plants and delayed appearance of negative effects connected with damage or death [86]. These results suggest that the vacuolar Na^+^ compartmentalisation plays a beneficial role in improving cereals’ salt tolerance.

Another element involved in the response to salinity is the family of high-affinity K^+^ transport (HKT) proteins, which, contrary to their name, are Na^+^ transporters (class 1) or Na^+^/K^+^ symporters (class 2) (Figure 1). HKT1 proteins remove Na^+^ from xylem sap and sequestrate Na^+^ into xylem parenchyma cells. The function of this mechanism is to confine toxic Na^+^ to the roots, therefore, it prevents the accumulation of Na^+^ in shoots and leaves, protecting the photosynthetic tissues from damage [58,87]. By contrast, SOS1 plays a role in the protection of the root since it exports Na^+^ out of the root and facilitates its loading into the xylem. These two mechanisms function antagonistically, and it is not fully understood how they are activated and regulated to avoid Na^+^ loading and unloading. The role of HKT proteins differs between species in response to salinity. For most species, Na^+^ exclusion from the leaf blade is correlated with enhanced salinity tolerance and is due to HKT1. The comparison of two rice varieties with different sensitivity to salinity showed that, under salinity stress, the Na^+^ concentration in the leaf blades was much lower in Ouukan383 (salinity tolerant) than in Kanniho (salinity sensitive). It is the result of a high expression level of *OsHKT1;4* in the leaf sheaths of in Ouukan383 cultivar, corresponding to higher Na^+^ accumulation in the leaf sheaths and lower Na^+^ accumulation in the leaf blades. What is more, under salinity conditions, the expression of the *OsHKT1;5* gene was induced in the roots of Ouukan383 but was repressed in the roots of Kanniho. These findings indicate that the expression of *OsHTS1s* might be correlated with better tolerance to salt stress [88]. In addition, a mutation in *OsHKT1;5* in rice showed that lack of OsHKT1;5 protein in roots leads to excess Na^+^ accumulation in leaves in response to salt stress [89]. On the other hand, the expression of *ZmHKT1;5* in two maize genotypes (*Zea mays* L.), SC131 (more tolerant) and SC132 (less tolerant), was not significantly affected under salt stress. However, the expression of *ZmHKT2* was highly induced in SC132 while its transcripts were absent in SC131. It can be concluded that differences in the salinity tolerance in these maize genotypes might be the result of weaker Na^+^ and K^+^ translocation to the shoots due to high expression of *ZmHKT2* in the roots of SC132 since it is responsible for reduced leaf K^+^ concentration, enhanced Na^+^ uptake in the roots and later more translocation to the shoots [90].

Signalling through the MAPK cascade leads to cellular responses against various stresses. This pathway relies on successive phosphorylation reactions, thus maintaining proper cell phosphorus (P) content is crucial. During salt stress, Cl^–^ may reduce plant P content due to ionic competition. Therefore, salinity may negatively affect the MAPK pathway. However, activation of the components of this signalling cascade does not always function as a positive regulator in the stress response. Hao et al. [91] showed that wheat TaMPK4, one of the members of MAPK, was a positive regulator in salt stress response. Sense- and antisense-expressing of *TaMPK4* in tobacco strongly modified plant growth under salinity. *TaMPK4*-overexpressing plants were much larger and showed a larger dry mass, leaf number and leaf areas, while *TaMPK4*-knockout plants were much smaller and showed a lower dry mass, leaf number and leaf areas, compared to WT plants. What is more, under salinity, plants with overexpression of *TaMPK4* had higher K^+^ and osmolyte contents and lower Na^+^ content than the WT plants, unlike *TaMPK4*-knockout plants [91]. Similarly, Arabidopsis plants with overexpression of *ZmSIMK1*, maize MAPK member, had increased tolerance to salt stress. Seeds of transgenic lines germinated better on medium containing NaCl, as well as at seedling stage, their growth was not inhibited, as was observed in WT plants [92]. On the other hand, the overexpression of wheat *TMKP1*, mitogen-activated protein kinase phosphatase (MKP), which is a negative regulator in the MAPK signalling pathways in Arabidopsis, resulted in improved tolerance to NaCl. Seeds of transgenic plants had a better germination rate and seedlings had lower content of MDA and ROS compared to WT. Improved resistance to salt stress in *TMKP1*-overexpressing plants was correlated with increased antioxidant enzyme activities, which resulted in less damage to cell components [93]. Additionally, Seong-Kon et al. [94] showed that rice *OsMAPK33* could play a negative role in salt tolerance. The expression of *OsMAPK33* was down-regulated until 8 h after the induction of salt stress, indicating that this is a negative regulator in response to salinity. Moreover, the overexpression of *OsMAPK33* in rice enhanced sensitivity to salt stress. It was assumed that it was a consequence of disrupted ion homeostasis since transgenic plants had reduced expression of ion transporter genes, such as the K^+^/H^+^ antiporter [94].

It was also reported that H_2_S might be an important player in plants’ response to salinity. It was shown that exogenous application of H_2_S improved salt tolerance in some cereals such as rice [95], wheat [96] and barley [97]. The protective role of H_2_S was the result of maintaining ion homeostasis, as well as reducing oxidative stress, which was reflected in decreased ROS and MDA contents under salt stress. In addition, antioxidant enzyme activity was increased with H_2_S application. Exogenous H_2_S might also enhance photosynthetic capacity as well as improve primary and energy metabolism. As it was shown in rice under influence of exogenous H_2_S, proteins related to glycolysis, tricarboxylic acid cycle and ATP synthesis were up-regulated in salt-treated plants [95]. Moreover, exogenous H_2_S up-regulated transcript level of genes encoding proteins involved in the SOS pathway and the MAPK pathway, as was recently shown in wheat [96].

## 4. Heavy Metals

The impact of heavy metals (HMs) on plants depends not only on the concentration and type of xenobiotic elements but also on their availability to plants, which is related to such soil factors as pH, cation exchange capacity, organic matter content and adsorption by clays. HMs in high concentration affect membrane permeability, inhibit enzymes activity, inactivate photosystems and disturb mineral metabolism [98]. Furthermore, HMs cause secondary oxidative stress, which results in the oxidation of plant membranes, damage of nucleic acid, leading to mutations, oxidative modifications of proteins resulting in loss of their activity, disruption of pigment function, and finally, cell death [99]. The toxicity of a specific substance, including HMs, depends on a variety of factors, e.g., how much of the substance organisms are exposed to, how they are exposed and for how long. Understanding the mechanisms underlying plant resistance or tolerance of plants to abiotic and biotic stress factors is extremely important in the era of global warming, where the mobility of pollutants in the environment increases [3]. However, some HMs are necessary (in non-toxic concentrations) for the proper development and growth of cereals. This category includes, among others, copper (Cu), iron (Fe), cobalt (Co), zinc (Zn), molybdenum (Mo), manganese (Mn), boron (B) and nickel (Ni), the presence of which is required for the proper functioning of the plant. However, excessive concentrations of even these essential micronutrients can also stress the plants. There is also a group of particularly highly toxic HMs including Pb, Hg, As and Cd that are ranked as the first, second, third and sixth, respectively, in the list of the US Agency for Toxic Substances and Disease Registry (ATSDR) [100].

HMs negatively affect the plant cell on many levels. They can directly inhibit enzymes and cause an oxidative burst, leading to the overproduction of ROS and RNS, which changes the oxidative potential in the cell [101]. ROS and RNS not only damage proteins, which can lead to their degradation but also alter membrane permeability, which puts the integrity of the cell at risk. In addition, HMs can induce chloroplasts and mitochondria damage, which inhibits basic metabolic processes in the cell, such as photosynthesis and the respiration chain. What is more, HMs also influence the stomatal movements and subsequently affect the transpiration rate [102]. HM also caused damage to DNA and inhibition of transcription and translation, which hinders the synthesis of proteins that may be of fundamental importance in the survival of the cell. All these changes lead to the failure of cell division, which prevents the correct growth and development of crops [98]. However, it should be observed that each of the HMs can affect the plant in a slightly separate way. Cd causes a strong inhibition of cereal growth, browning of the roots and chlorotic changes in leaves. Cd particularly affects photosynthetic enzymes such as Fe (III) reductase. In turn, Hg blocks the flow of water in the plant by interacting with the water channels, thereby blocking them. The action of Pb focuses on changing the permeability of the cell membrane, disturbing the hormonal balance of the plant and inhibiting the activity of selected enzymes due to the interaction of Pb with their sulfhydryl groups. As has a similar effect on enzymes, as it also reacts with sulfhydryl-containing proteins, disrupting their function. As also binds to vicinal thiols present in dehydrogenases, which not only inhibits cellular respiration but also leads to overproduction of ROS [103].

Due to the different effects of individual HMs on cereals, the response of the plant to HM stress is multifaceted and is associated with the activation of several signalling pathways causing a change in the expression of the relevant TFs and/or genes: (a) calcium-dependent signalling; (b) signalling mediated by MAPK; (c) signalling via ROS; (d) hormone signalling [9]. Calcium signalling occurs through several sensors which include calmodulins (CaM), calmodulin-like proteins, calcineurin B-like proteins and CDPK. The activation of individual sensors depends on the concentration of Ca^2+^. It was observed that both the recurrent and long-term Cr (VI) stress in rice increased the activity of CDPK [104]. The signalling cascade based on MPAK caused the phosphorylation of selected transcription factors (ABA-responsive element; ABRE, dehydration-responsive element binding; DREB, bZIP, MYB, MYC, NAC and WRKY-containing a conserved WRKYGQK domain and a zinc finger-like motif) resulting in the altered expression of genes related to the HM stress response [9]. Induction of OsMAPK2 and myelin basic protein kinase was recorded in Cd-treated rice. In response to the increased production of ROS, cereals improve the activity of their antioxidant system by increasing both enzymatic (SOD, CAT, APX, dehydroascorbate reductase) and non-enzymatic (betaines, proline and ascorbate) activities, which allows them to avoid or reduce oxidative damage to the plant cell, however, some redox imbalance is necessary for the induction of a proper stress response [105]. ROS and kinase-related pathways may cross with each other. In rice, the activation of MPAK by excessive accumulation of ROS was reported as a result of secondary oxidative stress induced by HM stress. What is more, ROS also influence changes in the plant’s hormonal system, in particular, auxin (AUX), ET and JA and ABA signalling. Treatment of rice with JA was shown to increase the antioxidant response of rice to Cd [106]. Treatment of rice plants with As resulted in a change in ABA metabolism, which influenced the modulation of signal transduction and the plant defence stress response [107]. Besides those signal transduction pathways, miRNAs also play a crucial role in the response to HM stress. miRNAs are 20–24 nucleotide non-coding RNAs that regulate gene expression at the post-transcriptional level by targeting mRNA degradation or by translation repression [108]. Due to the different properties of individual HMs, their uptake pathways, as well as signal induction and transmission, differ from each other.

As (V), being the main form of As in the soil, is similar in structure to P ions and thus its uptake into the plant is possible via phosphate transporters. Under anaerobic conditions, As also reaches the cell via aquaporins (AQPs). AQPs include various family subclasses of proteins that can uptake As, including tonoplast intrinsic proteins, cell membrane intrinsic proteins, and nodulin-like proteins. In rice, As (III) ions can be taken up by silicon pathways and methylene forms of low silicon transporter proteins (Lsi1 and Lsi2), which have the ability to transport As (III) ions both from and into the cell [109]. Due to the similarity of As (V) to P ions, ATP synthesis in plant cells is disturbed. As (III) in turn reacts with thiol groups of proteins, including enzymes, leading to the disturbance of cell homeostasis. In rice, As caused the production of ROS and the activation of the MAPK-inducing phosphorylation cascade including MKK4, MPK3, MPK4 [110] and calcium-dependent signalling by CaM, CaM kinase and CaM-like protein [107,111]. Moreover, rice induces down-regulation of miR172 (miRNA) and up-regulation of miR393, miR397 and miR408. The last one (miR408) has a direct role in targeting Cu-containing proteins or SOD [112]. On the other hand, ROS down-regulated miR397 targeted laccase, which led to increased activity of the lignin biosynthesis pathway by the accumulation of laccase enzymes [113]. Additionally, one of the miRNAs, miR528, was crucial for As tolerance in rice [114].

Cd in the environment occurs in an ionic form (Cd^2+^) and is bound into chelates. Cd^2+^ is taken up into the plant by non-specific HM transporters, whose levels depend on transpiration. The most important uptake routes for both Cd forms include Zn-regulated transporters, Fe-regulated transporters, hyperpolarisation-activated Ca^2+^ channels, depolarisation-activated Ca^2+^ channels, voltage-insensitive cation channels, yellow-stripe 1-like proteins (YSL) and the natural resistance-associated macrophage protein (NRAMP). Transport to the xylem occurs via apoplastic ATP-binding cassette (ABC) transports and P1B ATPase and H^+^/Cd^2+^ antiports. During defence responses, cereals activate TFs such as DREB, APETALA2 (AP2) and bZIP [103]. Cd accumulation activated the MAPK pathway: MAPK2, MPK3, MPK6, MSRMK3, WJUMK in rice [110,115,116] and MPK3 [49] in maize. It also activated components of the hormonal pathway, mainly by auxins: MAPK3/6/7, YUCCA, PIN proteins, ARF (auxin response factors) and IAA [117]. Exposing rice to Cd stress led to the up-regulation of miR441, and down-regulation of 12 other miRNAs, including miR192, which targeted ABC transporters. Increased activity of ABC transporters enables Cd sequestration and stress alleviation [118]. Cd up-regulated the transcription factors belonging to MYB, AP2, DREB, WRKY and NAC at different time intervals in rice [119]. As for MYB, OsMYB45 was especially related to Cd toxicity, as its mutation increased H_2_O_2_ content in the leaves of mutant and decreased CAT activity compared to the WT plants [120], and OsARM1 (arsenite-responsive MYB1) regulated As-associated transporters genes *OsLsi1, OsLsi2* and *OsLsi6* [121].

In most plants, the occurrence of aluminium (Al) is limited to the roots, although the presence of Al-citrate in the xylem and Al-oxalate in the leaves of buckwheat was reported [122]. Al is excluded into the soil by organic acids aided secretion through transporters such as the Al-activated malate transporter (ALMT) family, ABC transporters family (STAR1 and STAR2), multidrug and toxic compound extrusion (MATE) family and aluminium transporter 1 (NRAMP/NRAT1) family [123]. In wheat, Al accumulation enabled pathways dependent on MAPK: 48 kDa MAPK, 42 kDa protein kinase [124], Ca: myosin, calpain, phospholipase C, phospholipase A2 [125], and ethylene: ALMT1, 1-aminocyclopropane-1-carboxylic acid (ACC) synthase (ACS), ACC oxidase (ACO) [126]. Similar to previously described HMs, Al also down-regulated most of the miRNAs in rice such as miR156, miR395, miR398, miR159 and only miR399, miR166, miR168 were up-regulated in response to Al [127]. This however is not true for all crops, as maize showed mostly miRNAs up-regulation with the exception of miR171 and miR396 [128]. MiR395 targets genes of ATP sulfurylase (APS) and SULTR2:1, which are crucial for GSH and phytochelatin (PCs) synthesis [129].

Another important HM is Hg. The bioavailable Hg compounds in the soil are Hg^2+^ and methylmercury. Hg with a hydrophilic character is easily trapped by the roots, transported to the shoots, and then released back into the atmosphere in gaseous form. Hg tends to accumulate in the roots and cannot be transferred to plant shoots. Transport of Hg in the plant is possible due to ABC transporters. They can pump Hg^2+^ conjugates to or from the vacuole of higher eukaryotes [130]. It was shown that an accumulation of Hg led to the activation of MAPK proteins in rice, especially MSRMK2, MSRMK3, WJUMK [115], and the ET pathway via OsACS2, OsACO1, OsACO2, OsACO5 and OsACO6, 5 MAPKKK, 1 MAPKK and 2 MAPK [131].

Pb in the form of a dipositive cation is passively aborted by root hairs. Its further transport is severely limited by its low solubility. Pb transport in the plant is accomplished by the apoplast of xylem tissues but is blocked in the Kasparian bands of the endoderm. It can then be sequestered via ABC transporters, P-type pumps, pleiotropic drug resistance (PDR1), inner membrane proteins of mitochondria, ATM1, leucine-rice repeat proteins (LRR), Ca^2+^ gated channels, cyclic nucleotide ion gated channels and K^+^ gated channels [132]. In rice, Pb activates 34 kDa, 40 kDa and 42 kDa MAPK, and a calcium-dependent pathway via CDPK-like kinase [133].

In order to limit the negative effects of HMs, the signal cascade causes adaptive changes in the plant cell, relying on detoxification to prevent the involvement of HMs in undesirable toxic reactions. Defence strategies include preventing or reducing the uptake by limiting the transport of metal ions to the apoplast by binding them to the cell wall or cell exudate, or by inhibiting long-distance transport [134]. To achieve that, activation of appropriate TFs and induction of the transcription of particular genes related to the HMs response is necessary. Some of the up-regulated genes are associated with the activation or amplification of selected signal transduction pathways. For example, As treatment of rice increased the expression of the ABA biosynthesis genes: *OsNCED2* and *OsNCED3* [135], while chromium treatment of rice increased the expression of four ET biosynthesis-related genes (*ACS1*, *ACS2*, *ACO4* and *ACO5*) [136,137], two genes associated with MAP cascades (*OsMPK3*, *OsCML31*), three protein kinase-related genes (*OsWAKL-Os*, *OsLRK10L-2*, *OsDUF26-If*) and two TF-related genes (*OsWRKY26*, *OsAP2*/*ERF-130*) [137]. Another group of genes expressed by the action of HMs are genes encoding phosphatases. Phosphorylation/dephosphorylation is the most common post-translational modification, whose role is to activate and deactivate selected proteins, which results in the adaptation of the metabolism to the plants’ needs. In rice treated with chromium, increased expression of five families of genes encoding phosphatases (*OsLMWP*, *OsDSP*, *OsPP2A*, *OsPTP* and *OsPP2C*) was observed [137]. Due to the fact that one of the strategies for reducing the negative impact of HMs is their translocation, another group of genes up-regulated as a result of stress are those related to the transport of HMs. In rice, Cr strongly induced a number of genes involved in the vesicle trafficking pathway, including five OsExo70 genes (*Os01g0763700*, *Os06g0255900*, *Os01g0905300*, *Os01g0905200* and *Os11g0649900*) and one *Tom1* gene (*Os05g0475300*) [137]. In durum wheat, the exposure to Cd induced several vacuolar HM transporter genes, especially *ZIF1*, *ZIF-like* genes [138].

When HMs are present at elevated concentrations, cells activate a complex network of storage and detoxification strategies, such as chelating metal ions with phytochelatins (PC) and metallothioneins (MTs) in the cytosol, as well as transport and sequestration into the vacuole via vacuole transporters [139]. HMs activate the synthesis of phytochelatin synthase (PCS) and metallothionein, and then HM–PC and HM–MTs complexes of low molecular weight (LMW) are formed in the cytosol. LMW HM–PCs complexes are consistently transported across the tonoplast into the vacuole via the ATP binding cassette and the V-ATPase transporter (ABCC1/2). After compartmentalisation, the LMW complexes further integrate HMs and are generated by chloroplasts sulfide (S^2−^) to eventually form HM–PC complexes of high molecular weight (HMW). MTs regulate cellular redox homeostasis independently and by stimulating the antioxidant system and stabilising high cellular GSH concentrations. It was well documented that the biosynthesis of PCs can be regulated at the post-translational level by metals in many plant species. However, the overexpression of the phytochelatin synthase (PCS) gene in plants does not always result in enhanced tolerance to HM stress [140]. Moreover, MTs not only bind HM but also partake in the elevation of oxidative stress by acting as ROS scavengers, thus, integrating those two pathways [141]. MTs are tissue-specific. For example, the *OsMT2c* gene encoding for type 2 MT was expressed in the roots, leaf sheathes and leaves of rice, but was almost absent in seeds [142]. Moreover, to protect proteins against HM stress, HSP proteins are also synthesised, belonging to HSPs70, HSPs60, HSPs90, HSPs100 and HSPs classes. HSPs70 were induced in rice by As, Ag, Cu, Cd and Cr (HSP70, BiP), HSPs60s by Hg (cpn60^2^), HSPs90 by Cu, As and Cd (HSP81-2, HSP82, HSP81-1), HSPs100 by As, Cu and Co (HSP101, ClpB-C), and HSPs by Cu, Cd, Fe, Al and Zn (HSP17.4, HSP23.9, HSP78.3) [140].

## 5. Biotic Stress

Plants are exposed to a wide variety of pathogens and pests, the life cycle of which and the impact on plants differ significantly. Therefore, it is difficult to identify one common signalling pathway associated with the biotic stress response. The plant–parasite relationship is quite specific and depends on both the defence mechanisms and the structure of the plant itself, as well as those of pathogen, therefore the signal transduction pathway is multifaceted and quite strongly individualised. Research on the subject is fairly limited, but in this review, we attempted to describe its known elements.

Plants have an innate immune system able to recognise evolutionarily conserved microbe/pathogen-associated molecular patterns or herbivore-associated molecular patterns [143,144]. The presence of transmembrane pattern recognition receptors and intracellular proteins of the nucleotide-binding domain and leucine-rich repeat superfamily enables the identification of pathogens/herbivores by plant cells which leads to induction of defence reactions including the synthesis of signalling molecules such as SA, ABA, JA, ET, H_2_O_2_ and NO [145]. The activation of those signalling patterns can cause alterations in gene expression, leading to specific defence responses. Both pathogens and insects can act locally and systematically [145].

Plants launch defence responses to shield themselves against pathogens and pests. Those responses are regulated by the infestation-induced production of hormones. SA, JA, ET and ABA are vital players in induced mechanisms against biotic stresses [146]. SA-dependent responses are usually efficacious against biotrophs, while JA-dependent responses are successful against necrotrophs and phytophagous insects [147]. Defence signalling of SA depends on the transcriptional co-factor called non-expresser of pathogenesis-related gene 1 (NPR1), ultimately leading to the activation of anti-microbial pathogenesis-related (PR) genes [148]. Following pathogen infection/insect infestation, molecules such as ABA, JA, SA, ET, H_2_O_2_ and NO are accumulated at different time points and convergence of signalling pathways can occur in a plant [149,150].

The biotrophic barley powdery mildew *Blumeria graminis* and the hemibiotrophic *Bipolaris sorokiniana* are economically significant pathogens of *H. vulgare*. To assess the barley defence responses to these pathogens, alternations in SA and genes of SA-dependent responses (*PR1*, *PR2*, *PR3* and *PR5*) were studied, which revealed that the level of SA was significantly enhanced in infected barley plants (both resistant and susceptible) at 24 h post-inoculation compared to control plants. Furthermore, time-course experiments showed a clear contradiction in patterns of expression of SA-dependent genes upon barley inoculation with *B. graminis* and *B. sorokiniana.* These studies also showed that the expression of *PR1* and *PR2* genes was induced in resistant barley inoculated with *B. sorokiniana* contrary to *B. graminis* infestation, indicating different SA-dependent responses in barley plants infested with fungal pathogens with different lifestyles [2].

MYB transcription factors play a vital role in cereal plant defence including responses to fungal pathogens. Wei et al. [151] presented results on characterisation of the *TaPIMP2* gene encoding a pathogen-activated MYB protein in *T. aestivum*. The expression of *TaPIMP2* was altered to a different extent and speed upon inoculation with *B*. *sorokiniana* or *Rhizoctonia cerealis*. In addition, different expression patterns of *TaPIMP2* were observed after *T. aestivum* plants were sprayed with ABA, 1-aminocyclopropane-1-carboxylic acid (ACC, precursor of ethylene) or SA. Silencing of *TaPIMP2* decreased the resistance of *B*. *sorokiniana*-resistant wheat to *B*. *sorokiniana* infection but did not change the resistance of *R*. *cerealis*-resistant wheat to *R*. *cerealis* infection. On the other hand, the overexpression of *TaPIMP2* remarkably increased resistance to *B*. *sorokiniana* rather than *R*. *cerealis* in transgenic wheat. Moreover, it was observed that TaPIMP2 is engaged in wheat resistance to *B*. *sorokiniana* due to stimulation of the expression of *PR1a*, *PR2*, *PR5* and *PR10*.

After the plant is mechanically injured or infested with necrotrophic pathogens or insects, the accumulation of JA and its derivatives—oxylipins (called jasmonates)—occurs [152]. For example, infestation of maize with a lepidopteran pest, the beet armyworm caterpillars (*Spodoptera exigua*) induced synthesis of JA, MeJA and jasmonoyl-L-isoleucine in infested-maize leaves [153]. There are two separate branches of the JA signalling that have a negative influence on each other: the ERF branch and the MYC branch [154]. The ERF branch is induced upon infestation with necrotrophs and is controlled by the AP2/ERF-domain transcription factors such as ERF1 and octadecanoid-responsive AP2/ERF 59 (ORA59). Furthermore, the ERF branch is co-regulated by ET and triggers the expression of many ERF-branch genes including the marker gene encoding plant defensin 1.2 (PDF1.2) [155]. Dong et al. [156] identified and characterised *B*. *sorokiniana*-induced defence gene (*TaPIEP1*) from the ERF branch (B-3c subgroup) of wheat. The mRNA level of *TaPIEP1* was induced upon both inoculations with *B*. *sorokiniana* and treatments with ET, JA, and ABA. Transgenic *T*. *aestivum* plants overexpressing *TaPIEP1* showed enhanced resistance to *B*. *sorokiniana*. The increased resistance of transgenic wheat lines showed also increased transcript levels of defence-associated genes from the ET/JA pathways. Wheat is one of the main cereals crucial for food production worldwide, therefore its pathogens should be one of the main focuses in biotic stress studies. Besides *B*. *sorokiniana*, *Puccinia striiformis*, which also causes stripe rust, is an important wheat pathogen. In response to *P*. *striiformis* reactive oxygen species burst is observed. Early accumulation of ROS leads to an increase in chlorophyll a and b levels, as well as to activation of antioxidative enzymes. It contributes to plant resistance to this pathogen [157].

Jisha et al. [158] proposed a model for the role of the AP2/ERF transcription factor, OsEREBP1, during the response of rice plants to infection with the bacterium *Xanthomonas oryzae* pv. *oryzae*. The authors suggested that enhanced expression of *OsEREBP1* can lead to accumulation of JA, which mediates activation of the helix–loop–helix transcription regulator RERJ1 and induces linalool synthase activity so that volatile monoterpene linalool molecules are accumulated resulting in improved tolerance to *X. oryzae* pv. *oryzae* infection.

The brown planthopper (*Nilaparvata lugens*) is a hemipteran pest infesting rice plants. This insect injures plants through feeding, and it also transmits rice grassy stunt virus and rice ragged stunt virus [159]. Xylanase inhibitors were described as players participating in plant defence. Zhan et al. [160] presented that infestation with *imagines* of *N. lugens*, wounding or MeJA treatment increased transcript and protein levels of OsXIP (an XIP-type rice xylanase inhibitor). By studying 5′ deletion in *OsXIP* promoter in rice mutant plants invaded by *N. lugens,* a 562 bp region was shown as crucial for the response to stress induced by pest feeding. Furthermore, a basic helix–loop–helix protein (OsbHLH59) and an AP2/ERF-transcription factor OsERF71 directly reacted with 562 bp sequence to induce the expression of *OsXIP*. The expression of genes *OsbHLH59* and *OsERF71* was also stimulated in rice roots and shoots by wounding and submerging in MeJA.

Fusarium head blight induced by *Fusarium* species such as *F*. *graminearum* is a globally important fungal disease of wheat. Transcriptional profiling of moderately resistant and susceptible to *F*. *graminearum* winter wheat cultivars have shown 2169 differentially expressed genes, induced by jasmonate and ethylene, e.g., encoding thionin, lipid-transfer protein, defensin and GDSL-like lipase. Moreover, defence-activated genes encoding jasmonate-dependent proteins were up-regulated in response to infection with *F*. *graminearum,* such as, for example, the subfamily of mannose-specific jacalin-like lectin-containing proteins [161].

During an infestation, pathogens and pests secrete effectors into host plant tissues. These effectors interact with plant defence systems, which may lead to effective colonisation and the spread of the infection [162]. Darino et al. [163] performed functional characterisation of the biotrophic fungus *Ustilago maydis* (causing smut disease on maize plants) effector jasmonate/ethylene signalling inducer 1 (Jsi1). Jsi1 reacts with members of the plant corepressor protein family Topless/Topless-related (TPL/TPR). It was shown that the increased expression of *Jsi1* in maize led to activation of the ERF-branch pathway by an ET-responsive element-binding factor-associated amphiphilic repression (EAR) motif, which takes after EAR motifs from plant ERF transcription factors interacting with TPL/TPR proteins. Interestingly, phytopathogen effector candidates with EAR motifs were also found to be secreted by an ascomycete fungus *Magnaporthe oryzae* (affecting rice) and a Basidiomycota fungus *Sporisorium reilianum* (affecting maize and sorghum) [163].

In winter wheat field studies, it was shown that JA application induced resistance to cereal aphids (*Metopolophium dirhodum*, *Sitobion avenae*, *Rhopalosiphum padi*) and thrips (*Limothrips denticornis* and *Thrips angusticeps*). JA at first caused a significant decrease in the number of pests, which, even though it increased in time, remained lower on wheat treated with JA [164].

The MYC branch is induced upon mechanical injury or feeding by insects. This branch is controlled by basic helix–loop–helix leucine zipper transcription factors MYC2, MYC3 and MYC4, and it is also coordinated by ABA [165]. The MYC-branch activation results in the induction of JA-responsive gene expression including marker genes of the MYC-branch such as *vegetative storage protein 1* and *2* (*VSP1* and *VSP2*) [166].

The rice water weevil (*Lissorhoptrus oryzophilus*) is the most harmful coleopteran pest of *O*. *sativa* plants. It was proven that the treatment of rice seeds with jasmonates led to resistance against *L. oryzophilus* but rice growth and fitness were reduced. Jasmonates caused delayed emergence and heading, and after full development of plants, lower yield in comparison to plants grown from untreated seeds [167,168]. Therefore, it can be stated that plant fitness is decreased upon activation of JA-dependent defence responses, however, other hormones including ABA, SA, GAs, AUX and BRs are also substantial regulators of the immune–fitness balance caused by phytopathogens [169,170]. In addition, the decrease in plant growth elicited by JA is most probably regulated via signalling crosstalk with AUX, SA, BRs, GAs and CKs [171].

The crosstalk between hormonal pathways promotes the induction of efficient responses against pathogens and pests [172]. Many observations of the mutual interaction between the SA and JA pathways were made [173]. Pharmacological studies showed that the expression of *PDF1*.2 and *VSP2* is sensitive to SA treatment. The opposed influence of SA on JA-depended responses was observed. It was shown that exogenous treatment with SA decreased the expression of the JA-responsive genes (*PDF1*.2 and *VSP2*) activated by MeJA, the necrotrophic fungi *Alternaria brassicicola* and *Botrytis cinerea*, and the western flower thrips (*Frankliniella occidentalis*) and *P*. *rapae*. However, infestation with the biotrophic oomycete *Hyaloperonospora parasitica* leading to SA-activation defence antagonised MeJA-dependent expression of *PDF1.2* and *VSP2* and infection with *H*. *parasitica* diminished *P*. *rapae*-activated expression of *VSP2* [174]. Moreover, it was documented that this effect (induced by SA exogenous exposition) persists in the next plant generation [175]. The antagonism between SA and JA pathways can change resistance to biotic stressors. It was observed that activation of the SA signalling by exogenous exposition to SA or infestation with the hemibiotrophic bacterium *Pseudomonas syringae*, made the plants more susceptible to *A. brassicicola* [176,177]. Moreover, decreased SA responses in transgenic plants expressing a bacterial salicylate hydroxylase gene (*nahG*) and *npr1* mutant plants were interdependent with attenuated feeding by the cabbage looper (*Trichoplusia ni*) caterpillars [178].

Similar antagonism is present in the ERF and the MYC branch. For example, it was proved that inducing the MYC2 branch in plants inhibits the ERF branch activated by *P. rapae* feeding, hence they are less alluring to the herbivore. Moreover, caterpillars of *P. rapae* preferred to feed on *jin1* (MYC2 transcription factor) mutants and *ORA59*-overexpressing ones more than on WT plants, showing that the ERF and the MYC branch crosstalk changes host–insect herbivore interactions [154]. This antagonism between the ERF and the MYC branch can also change resistance to necrotrophic pathogens. The ERF branch was elevated in plants with *MYC2*-mutated *jin1* and ABA biosynthesis mutant (*aba2-1*), leading to increased resistance to necrotrophs (*B. cinerea*, *Plectosphaerella cucumerina*, *Fusarium oxysporum*) [166,179,180,181,182].

Vast crosstalk between hormonal signalling pathways permits the plant under biotic stress for precise regulation of defence responses at various levels of plant organisation [183]. As elicitation of parasite-inducible responses is not without metabolic cost, trade-offs between immune defence and growth and development are clearly noticeable in plant organisms [146,184,185,186]. Hormonal crosstalk is sometimes discussed as an evolutionary cost-limiting strategy. Some researchers argue that this crosstalk may have evolved as a countermeasure to lessen energy costs by retardation of ineffective defence responses against specific invaders [187,188]. This hypothesis also seems to be confirmed by Vos et al. [189]. The authors analysed the effect of hormonal crosstalk on biotic stress resistance and host fitness upon multi-species infestation. Activation of SA- or JA/ABA-mediated responses by the biotrophs *Hyaloperonospora arabidopsidis* or *P. rapae*, respectively, decreased the level of induced JA/ET-response against the following infestation with *B. cinerea*. Notwithstanding, although there was increased susceptibility to this second invader, no long-term negative consequences were observed on host fitness when plants had been infected by multiple parasites. The authors concluded that host hormonal crosstalk during multi-parasite interactions gives the plants an opportunity to put their defence in order of importance while decreasing the energy fitness costs linked to activation of immune responses. This issue is extremely interesting and requires further research, especially in the context of crop plants including cereals.

## 6. Crosstalk Signalling between Abiotic and Biotic Stress

**Figure 2:**
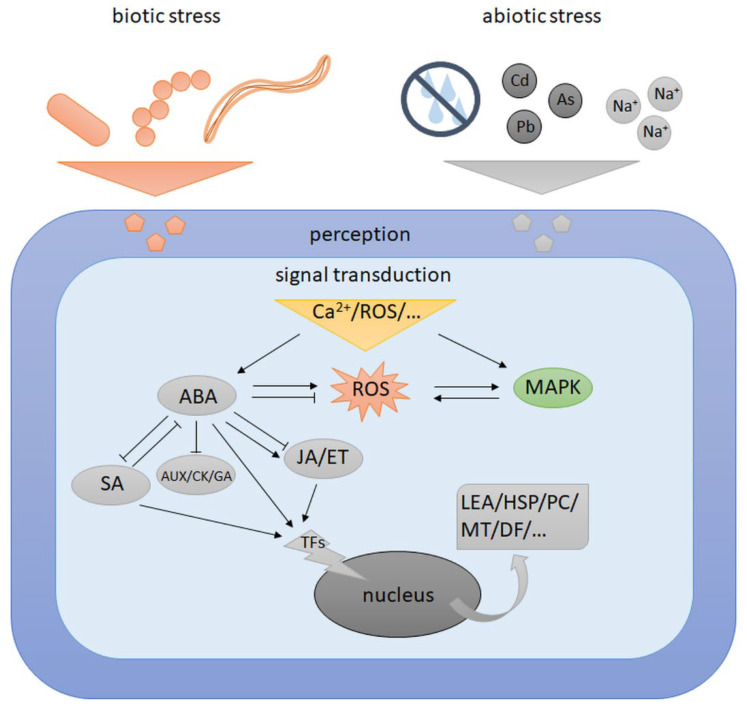
Scheme for the crosstalk signalling between abiotic and biotic stress. Both stress factors are first recognised by plant cells and then information is transduced through chemical signals such as Ca^2+^, reactive oxygen species (ROS), as well as mitogen-activated protein kinases (MAPK) cascades. Abscisic acid (ABA) is mostly involved in abiotic stress acclimation, while salicylic acid (SA) and jasmonate/ethylene (JA/ET) are responsible for the reaction to abiotic as well as biotic stresses. Finally, phytohormones up-regulate transcription factors (TFs), which then contribute to expression of genes related to stress response, e.g., late embryogenesis abundant proteins (LEA), heat shock proteins (HSP), phytochelatins (PC), metallothioneins (MT), defensis (DF).

Current research on biotic and abiotic stress response pathways in plants suggests that there are significant similarities between them. The responses of cereals plants to biotic and abiotic stress are a complex web of interactions between secondary messengers, ROS, phytohormones, antioxidants, photosynthetic pigments, secondary metabolites, protein kinases, TFs, photosynthesis efficiency and chlorophyll a fluorescence parameters and ultrastructural adjustments [190,191,192]. Plants subjected to abiotic stress, e.g., high temperature, drought, salinity, are often more sensitive to subsequent attacks by pathogens [193]. There are reports about the decrease in the disease resistance of crops due to high humidity and high temperature [194]. Both types of stress factors cause the increase in such parameters as Ca^2+^, ROS, and pH levels in the apoplast. MAPK kinases are activated, which is a common response to both stresses [195]. For example, OsMPK5 kinase in rice is an ortholog of AtMPK3 in Arabidopsis and NtWIPK in tobacco, which are well known to be activated by both different pathogens and abiotic environmental stimuli [195]. ABA triggered a signal and it negatively imposed on the signalling of defence hormones, e.g., SA. ABA/SA interaction is two-sided, as activation of SA signalling by pathogens lowers ABA concentration [194]. On the other hand, positive interactions were observed for JA/ET signalling in response to double stress. ABA can act as a molecular switch between both responses and plays a dominant role in the response to stress [196]. It can take place through the ABA-inducible genes *ERD15* and *ATAF1*, which may activate ABA-dependent biotic stress responses at the expense of abiotic responses [197]. A scheme for the interaction interface and overlapping signalling pathways of abiotic and biotic stress at the cellular level is presented in Figure 2.

CDPK families are also involved in crosstalk between biotic and abiotic stresses [198]. They are involved in various processes such as osmotic homeostasis, cell protection and root growth [44]. Some studies have reported that the CDPK genes not only behaved as positive regulators of abiotic or biotic stress signalling but also as negative regulators [44]. Overexpression of *OsCDPK12* in rice led to positive regulation of salt tolerance and negative regulation of blast resistance [199].

Phytohormones regulate the activity of transcription factors such as WRKY, MYB, ERF, NAC and the HSF family, which respond to both biotic and abiotic stress and play a vital role in the plant’s response to simultaneously occurring stresses. WRKY30 and WRKY13 have a dualistic function in response to drought, salinity, cold and pathogen attack in rice [200]. Some WRKY such as *OsWRKY76* antagonistically regulated the response of rice to blast disease and cold stress [201] but *OsWRKY82* improved resistance against pathogens and tolerance against abiotic stress via the JA and ET pathways [202]. The rice *OsWRKY45* is induced in response to ABA in various abiotic stress and also by infection with *Pyricularia oryzae* Cav. and *Xanthomonas oryzae* pv. *oryzae.* In a study by Qiu and Yu [203], it was shown that constitutive overexpression of the *OsWRKY45* led to a significant increase in the expression of PR genes, resistance to bacterial pathogens, as well as tolerance to salt and drought stresses.

MYB transcription factors also may be common element of the response to various stresses. The MYB factor TaPIMP1 from wheat confers tolerance to drought and salt and pathogens stress when overexpressed in tobacco [204]. Another one of the candidates for common TF for multiple stresses response is JAmyb. *JAmyb* expression in response to salinity and osmotic stress was observed in rice seedlings. Microarray analysis showed that *JAmyb* overexpression stimulated the induction of several defence-related genes, some of which are predicted to be involved in osmosis regulation, ROS removal and ion homeostasis [205]. Additionally, transgenic rice plants overexpressing *JAmyb* exhibited improved resistance to blast [206]. A study by Yokotani et al. [205], showed that *JAmyb* expression was induced by H_2_O_2_ and paraquat. However, it is known that ROS overproduction is a common response to biotic and abiotic stress and could overlap with other stress responses. It is suggested that *JAmyb* might play a role in the crosstalk between JA and ROS-signal transduction pathways in dual stresses [205].

Another important TF is NAC. NAC are plant-specific TFs induced in various developmental stages and under abiotic and biotic stress [207]. The enhanced expression of the *TaNAC4* gene in wheat was observed under the fungus, salinity, wounding and cold stress [208]. Expression of *OsNAC6* in rice was induced by abiotic stresses, including cold, drought and high salinity, as well as by biotic stresses, such as wounding and blast disease [207]. OsNAC6, among others, increases the activity of peroxidase, which elevates oxidative stress.

It was shown that genes encoding cold-responsive/late embryogenesis abundant (COR/LEA) proteins, participate in improving cold resistance and protection of cells from dehydration and low-temperature [209]. It is known that the ABA participates in the regulation of *COR* gene (*WRAB15* and *WRAB18*) expression in wheat. Studies by Talanova et al. [210] showed enhanced expression of *WRAB15* and *WRAB18* genes in wheat leaves caused by the Cd, hardening or their combination. This may indicate the participation of these genes in the protective and adaptive responses of plants to different stress factors [210].

As mentioned above, the effect of one stress can make plants more sensitive to the next stress. On the other hand, exposure of plants to one stress affects their response during the next stress leading to enhanced defence mechanisms to later stress. This phenomenon called “priming” results in a faster and stronger induction of basal defence mechanisms upon subsequent biotic stress factors [3]. “Metabolic memory” in higher plants requires less energy expenditure than defence directly induced by insect feeding or infection caused by pathogens.

A list of genes that may be crucial in signalling the response to biotic and abiotic stresses is given in Table 1 and Table 2.

## 7. Conclusions

Different stresses affect plants in various ways, therefore proper plant acclimation enabling plant survival is dependent on the crop’s ability to recognise the stress factor and its intensity, as well as on the ability to transmit the signal to the appropriate parts of both the cell and the plant in order to trigger an adequate response. While some plant defence mechanisms (such as ROS signalling) are not specific and occur under most stresses, others are strictly dependent on the specific stress factor (e.g., SOS). When cereals struggle to survive only with drought or with the presence of HMs, the situation is quite simple and well recognised in the literature. The problem appears when the same plant is affected by various stress factors at the same time or in short time intervals. In this case, the triggered defence mechanisms can be opposed to each other, which makes resistance to stress difficult. Therefore, learning about the signalling pathways and, more importantly, the interactions between them is crucial in plant cultivation, where multi-stress is common. It should be emphasised that these signal transduction pathways not only intersect with each other but are often opposed (ABA and SA), especially when both abiotic and biotic stress are present in the environment at the same time, which is of paramount importance for plant survival. By activating only selected response elements, and silencing others, it is possible to limit cereals’ energy expenditure on ineffective acclimatisation mechanisms. Reducing unnecessary energy consumption allows the plant to continue to develop and grow despite the presence of the stress factor, however, the same mechanism may lead to increase susceptibility to one stress when others occur. Therefore, in the near future, research should focus on signalling pathways crosstalk and multi-stress response.

## Figures and Tables

**Table 1:** The list of genes with a potential role under abiotic and biotic stress signalling pathways.

Gene	Plant	Changes in Expression Level/Physiological Effect	References
Drought stress			
*JERF1*	*Oryza sativa* L.	activates the expression of stress-responsive genes and increases the synthesis of the proline	[43]
*OsABA2*	*Oryza sativa* L.	increases ABA synthesis	[43]
*Os03g0810800*	*Oryza sativa* L.	increases ABA synthesis	[43]
*TaGST1*	*Triticum aestivum* L.	decreases ROSs	[33]
*ZmCCaMK*	*Zea mays* L.	participates in BR-induced antioxidant defence	[34]
*OsMKK1* *OsMKK4*	*Oryza sativa* L.	increases under drought	[48]
*OsMPK4* *OsMPK5* *OsMPK7* *OsMPK8*	*Oryza sativa* L.	increases under drought	[48]
*TaMKK1 * *TaMKKK16*	*Triticum aestivum* L.	increases under drought	[49]
*TaMPK8*	*Triticum aestivum* L.	increases under drought	[49]
*OsMKK10.2* *OsMPK3*	*Oryza sativa* L.	increases drought stress tolerance in rice via ABA signalling	[50]
*Dhn1* *Dhn3* *Dhn5* *Dhn7* *Dhn9*	*Hordeum vulgare* L.	increases under drought; show positive correlations with chlorophyll a and b contents; participates in osmotic adjustment; increases plant biomass and grain yield	[57]
*OsLEA3*-1 *OsLEA6*	*Oryza sativa* L.	enhances drought tolerance	[61]
*ZmPLC1*	*Zea mays* L.	partakes in interaction with other signalling pathways in guard cell; improves the drought tolerance	[62]
Salinity stress			
*SOS1* *SOS2* *SOS3*	*Triticum aestivum* L.	facilitates exclusion of toxic Na^+^ into root apoplast; significantly higher level in salt-tolerant genotype	[77]
*OsSOS1* *OsSOS2* *OsSOS3*	*Oryza sativa* L.	increases under salinity	[81]
*HvSOS1*	*Hordeum vulgare* L.	increases under salinity	[81]
*NHX1*	*Triticum aestivum* L.	increases under salinity	[82]
*HvNHX5*	*Hordeum vulgare* L.	increases under salinity in roots	[81]
*OsNHX1* *OsNHX2* *OsNHX4* *OsNHX5*	*Oryza sativa* L.	increases under salinity in roots	[81,85,86]
*OsHKT1;4* *OsHKT1;5*	*Oryza sativa* L.	increases salinity tolerance; decreases Na^+^ accumulation	[88,89]
*ZmHKT1;5* *ZmHKT2*	*Zea mays* L.	increases under salinity; reduces leaf K^+^ concentration; enhances Na^+^ uptake in the root; increases its translocation to the shoot	[90]
*OsMAPK33*	*Oryza sativa* L.	decreases under salt stress-negative regulator in salinity response	[94]
Heavy metals			
*OsMYB45*	*Oryza sativa* L.	decreases H_2_O_2_ content in the leaves; increases CAT activity	[120]
*OsLsi1* *OsLsi2* *OsLsi6*	*Oryza sativa* L.	participates in As transport	[121]
*OsNCED2* *OsNCED3*	*Oryza sativa* L.	increases ABA biosynthesis	[135]
*ACS1* *ACS2* *ACO4* *ACO5*	*Oryza sativa* L.	increases ET biosynthesis	[136,137]
*OsMPK3 * *OsCML31* *OsWAKL-Os * *OsLRK10L-2 * *OsDUF26-If * *OsWRKY26 * *OsAP2*/*ERF-130 * *OsLMWP * *OsDSP* *OsPP2A* *OsPTP * *OsPP2C*	*Oryza sativa* L.	increases under Cr toxicity	[137]
OsExo70 *(Os01g0763700* *Os06g0255900 Os01g0905300 Os01g0905200 Os11g0649900)*	*Oryza sativa* L.	increases under Cr toxicity; participates in vesicle trafficking pathway	[137]
*Tom1* (*Os05g0475300*)	*Oryza sativa* L.	increases under Cr toxicity; participates in vesicle trafficking pathway	[137]
*ZIF1* *ZIF-like*	*Triticum durum* Desf.	increases to Cd toxicity; participates in metal transport	[138]
*YSL2*	*Triticum durum* Desf.	increases to Cd toxicity; participates in metal transport	[138]
Biotic stress			
*PR1* *PR2*	*Hordeum vulgare* L.	increases expression under *B. sorokiniana* and decreases under *B. graminis* infestation	[2]
*Jsi1*	*Zea mays* L.	led to activation of the ERF-branch pathway by an ET-responsive element binding-factor-associated amphiphilic repression (EAR) motif	[163]
*OsEREBP1*	*Oryza sativa* L.	cause accumulation of JA	[158]
*OsERF71*	*Oryza sativa* L.	increases in roots and shoots as a result of wounding and submerging in MeJA	[160]
Multi-stress			
*OsWRKY76* *OSWRKY82*	*Oryza sativa* L.	antagonistically regulates the response of rice to blast disease and cold stress; increases resistance against pathogens and tolerance against abiotic stress via the jasmonic acid and ethylene pathways	[201,202]
*OsNAC6*	*Oryza sativa* L.	activates the expression peroxidase	[207]
*WRAB15* *WRAB18*	*Triticum aestivum* L.	increases under cadmium, hardening temperature, or their combination; protective and adaptive functions	[210]

**Table 2:** The list of mutants and transgenic plants with changed stress tolerance under abiotic and biotic stress.

Gene	Species	Type of Manipulation	Effect	Reference
*HVA1*	Barley	Overexpresion of *HVA1* in rice and wheat	Improves tolerance to water deficit	[59,60]
*TaSOS1-974*	Wheat	Overexpresion of *TaSOS1-974* in tobacco	Improves Na^+^ efflux and K^+^ influx rates in the roots, decreases oxidative damage of plasma membrane generated upon salinity	[78]
*TdSOS1∆972*	Durum wheat	Overexpresion of *TdSOS1∆972* in Arabidopsis	Increases water retention capacity and germination rate upon salinity	[79]
*TaNHX2*	Wheat	Overexpression of *TaNHX2* in *Solanum melongena* L. and *Helianthus annuus* L.	Increases salinity tolerance, improves growth, reduces ROS and MDA content	[83,84]
*HvNHX2*	Barley	Overexpression of *HvNHX2* in Arabidopsis	Improves growth under salinity	[85]
*OsNHX1*	Rice	Overexpression of *OsNHX1* in rice	Increases salt tolerance, delays appearance of negative effects connected with damages or death	[86]
*OsHKT1;5*	Rice	Mutation in *OsHKT1;5* in rice	Excesses Na^+^ accumulation in leaves under salinity	[89]
*TaMPK4*	Wheat	Overexpression of *TaMPK4* in wheat	Improves salinity tolerance, increases K^+^ and osmolyte contents and decreases Na^+^ content	[91]
*ZmSIMK1*	Maize	Overexpression of *ZmSIMK1* in Arabidopsis	Increases tolerance to salt stress	[92]
*TMKP1*	Wheat	Overexpression of *TMKP1* in Arabidopsis	Improves salinity tolerance, increases seeds germination rate, decreases ROS and MDA content under stress	[93]
*OsMAPK33*	Rice	Overexpression of *OsMAPK33* in rice	Enhances sensitivity to salt stress, disturbs ion homeostasis	[94]
*OsMYB45*	Rice	Mutation in *OsMYB45* in rice	Reduces resistance to Cd stress, increases H_2_O_2_ content, decreases CAT activity	[120]
*TaPIMP2*	Wheat	Overexpression of *TaPIMP2* in wheat	Increased resistance to *Bipolaris sorokiniana*	[151]
*TaPIEP1*	Wheat	Overexpression of *TaPIEP1* in wheat	Increased resistance to *Bipolaris sorokiniana*	[156]
*TaPIMP1*	Wheat	Overexpression of *TaPIMP1* in tobacco	Confers tolerance to drought, salt and pathogens stresses	[204]
*OsXIP*	Rice	Mutation in *OsXIP* in rice	Decreases response to stress induced by *Nilaparvata lugens*	[160]
*OsCDPK12*	Rice	Overexpression of *OsCDPK12* in rice	Increases salt tolerance, decreases blast resistance	[199]
*JAmyb*	Rice	Overexpression of *JAmyb* in rice	Improves resistance to blast	[205]
*OsWRKY45*	Rice	Overexpression of *OsWRKY45* in Arabidopsis	Increases resistance against pathogens and tolerance against abiotic stress	[203]

## Data Availability

Not applicable.
